# 2-Ferrocenyl-*N*-(6-methyl-2-pyrid­yl)benzamide

**DOI:** 10.1107/S1600536810008342

**Published:** 2010-03-13

**Authors:** John F. Gallagher, Steven Alley, Alan J. Lough

**Affiliations:** a School of Chemical Sciences, Dublin City University, Dublin 9, Ireland; b Department of Chemistry, 80 St. George Street, University of Toronto, Toronto, Ontario, Canada M5S 3H6

## Abstract

The title compound, [Fe(C_5_H_5_)(C_18_H_15_N_2_O)], a product of the reaction of 2-ferrocenylbenzoic acid and 2-amino-6-methyl­pyridine, crystallizes with two dissimilar mol­ecules in the asymmetric unit. In one mol­ecule, the picoline amide group is directed away from the 2-ferrocenylbenzene moiety (*anti*) whereas in the other, these are proximate (*syn*). In the crystal structure, mol­ecules aggregate into dimers *via* cyclic, asymmetric N—H⋯N inter­actions with graph set *R*_2_^2^(8), and are further augmented *via* intra­molecular C—H⋯O=C and inter­dimer C—H⋯π(arene) inter­actions. Dimers are linked into chains along the [102] direction *via* weak C—H⋯O hydrogen bonds.

## Related literature

For background information and related structures, see: Donnelly *et al.* (2008 ▶); Gallagher *et al.* (2008 ▶, 2009 ▶).
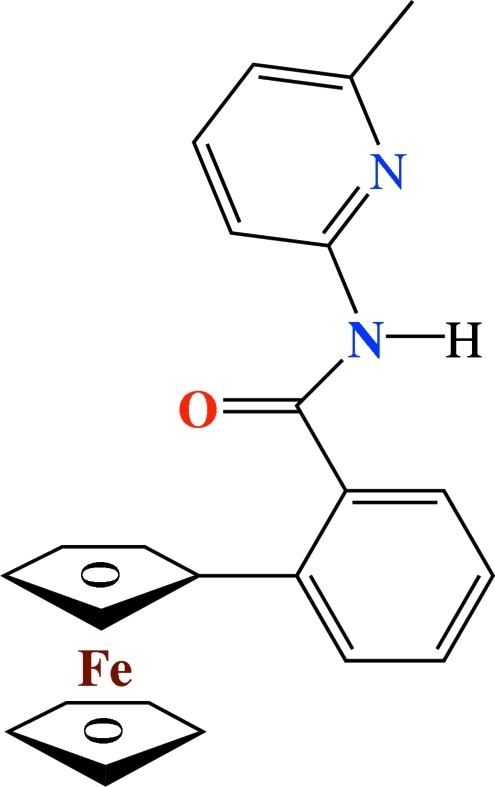


## Experimental

### 

#### Crystal data

[Fe(C_5_H_5_)(C_18_H_15_N_2_O)]
*M* *_r_* = 396.26
Trigonal, 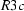

*a* = 22.8234 (9) Å
*c* = 36.9342 (15) Å
*V* = 16661.7 (11) Å^3^
*Z* = 36
Mo *K*α radiation
μ = 0.83 mm^−1^
*T* = 150 K
0.30 × 0.25 × 0.20 mm

#### Data collection

Nonius KappaCCD diffractometer
Absorption correction: multi-scan (*SORTAV*; Blessing, 1995 ▶) *T*_min_ = 0.788, *T*_max_ = 0.851
16925 measured reflections
6243 independent reflections
4606 reflections with *I* > 2σ(*I*)
*R*_int_ = 0.085

#### Refinement

*R*[*F* ^2^ > 2σ(*F* ^2^)] = 0.047
*wR*(*F* ^2^) = 0.099
*S* = 1.01
6243 reflections
490 parameters
1 restraint
H-atom parameters constrained
Δρ_max_ = 0.34 e Å^−3^
Δρ_min_ = −0.32 e Å^−3^
Absolute structure: Flack (1983 ▶), 2941 Friedel pairs
Flack parameter: 0.016 (18)

### 

Data collection: *KappaCCD Server Software* (Nonius, 1997 ▶); cell refinement: *DENZO-SMN* (Otwinowski & Minor, 1997 ▶); data reduction: *DENZO-SMN*; program(s) used to solve structure: *SHELXS97* (Sheldrick, 2008 ▶); program(s) used to refine structure: *SHELXL97* (Sheldrick, 2008 ▶) and *SORTX* (McArdle, 1995 ▶); molecular graphics: *PLATON* (Spek, 2009 ▶); software used to prepare material for publication: *SHELXL97* and *PREP8* (Ferguson, 1998 ▶).

## Supplementary Material

Crystal structure: contains datablocks global, I. DOI: 10.1107/S1600536810008342/tk2637sup1.cif

Structure factors: contains datablocks I. DOI: 10.1107/S1600536810008342/tk2637Isup2.hkl

Additional supplementary materials:  crystallographic information; 3D view; checkCIF report

## Figures and Tables

**Table 1:** Hydrogen-bond geometry (Å, °) *Cg*1 and *Cg*2 are the centroids of the C31*A*–C36*A*, C31*B*–C36*B* rings, respectively.

*D*—H⋯*A*	*D*—H	H⋯*A*	*D*⋯*A*	*D*—H⋯*A*
N1*A*—H1*A*⋯N6*B*	0.88	2.24	3.113 (5)	172
N1*B*—H1*B*⋯N6*A*	0.88	2.14	3.004 (5)	167
C3*A*—H3*A*⋯O1*A*	0.95	2.26	2.853 (7)	119
C3*B*—H3*B*⋯O1*B*	0.95	2.28	2.839 (7)	117
C7*B*—H7*B*2⋯O1*B*^i^	0.98	2.60	3.050 (6)	108
C7*A*—H7*A*1⋯*Cg*1	0.98	2.78	3.688 (7)	154
C7*B*—H7*B*1⋯*Cg*2	0.98	3.68	3.490 (7)	140

Symmetry code: (i) 
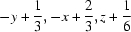
.

**Fig. 1.**
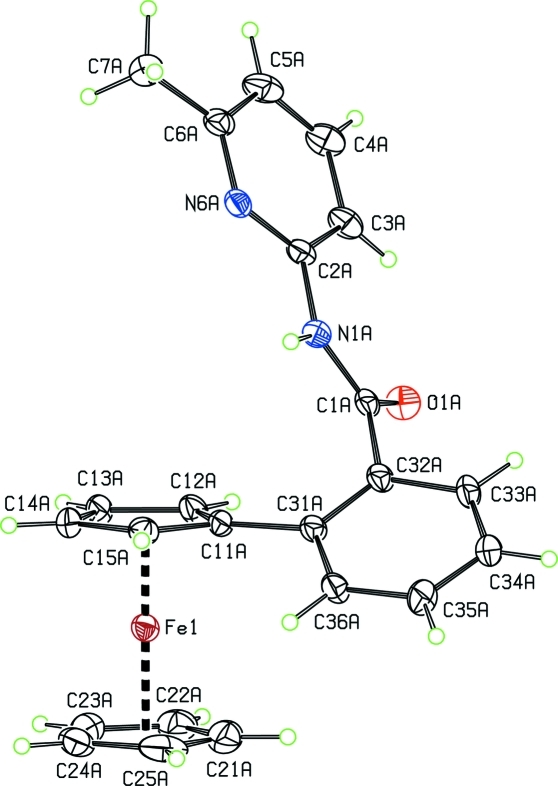
A view of molecule (A) in (I) with the atomic numbering scheme. Displacement ellipsoids are drawn at the 30% probability level.

**Fig. 2.**
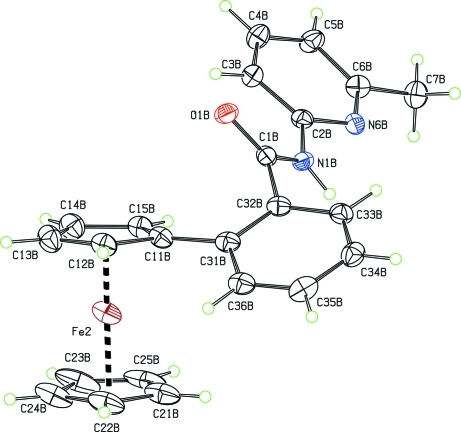
A view of molecule (B) in (I) with the atomic numbering scheme. Displacement ellipsoids are drawn at the 30% probability level.

**Fig. 3.**
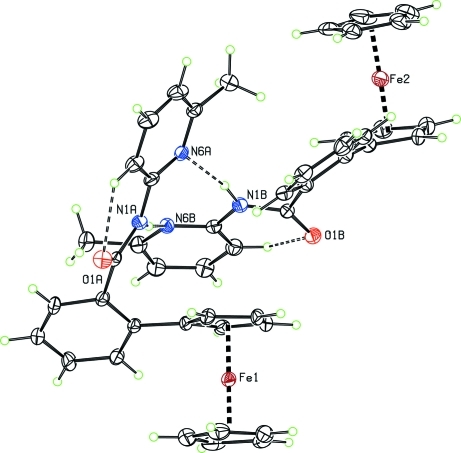
A view of the cyclic, asymmetric hydrogen bonded dimer in (I). Displacement ellipsoids are drawn at the 30% probability level. Intramolecular N–H···N and C–H···O contacts are shown as dashed lines.

**Fig. 4.**
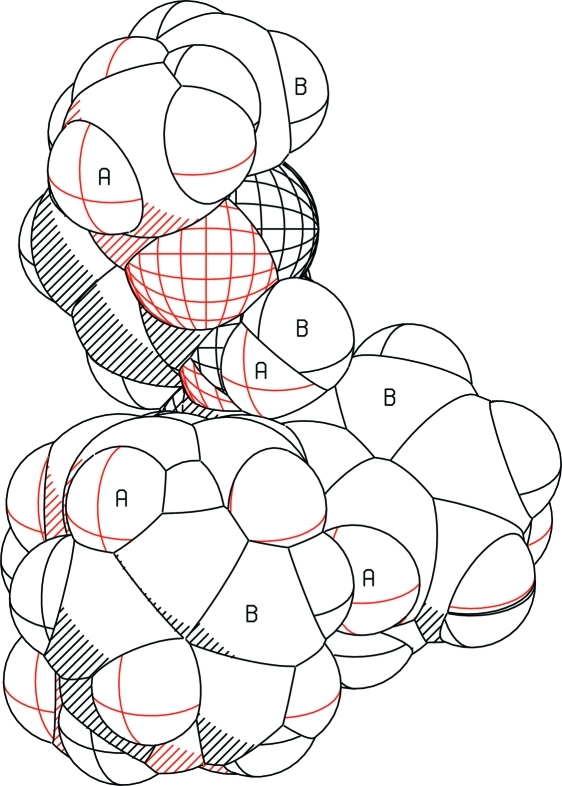
A best fit of molecules (A) and (B) with atoms drawn as their van der Waals spheres.

## supplementary crystallographic information

### Comment

The condensation reactions of benzoic acids and benzoyl chlorides with aromatic amino derivatives are well established. For those amines containing an ortho-ring N atom (such as ortho-aminopyridine), two products can be isolated having either the 1:1 or 2:1 benzoyl:pyridine components, and with yields and product ratios depending on the reaction conditions. Herein, we report the molecular and crystal structure of the ferrocene derivative, 2-ferrocenylbenzoyl-*N*-(3-methyl-2-pyridine), (I).

We have reported on the structure of a 1:1 derivative recently namely 2,3-difluoro-*N*-(2-pyridyl)benzamide (II) (Gallagher *et al.*, 2008) together with related fluoro(pyridyl)benzamides (Donnelly *et al.*, 2008) and an example of a 2:1 organometallic ferrocenyl analogue of these systems where a sterically bulky ferrocenyl group is a substituent on the aromatic ring namely, 2-ferrocenyl-*N*-(2-ferrocenylbenzoyl)-*N*-(5-methyl-2-pyridyl)benzamide (III) (Gallagher *et al.*, 2009).

Compound (I), a product of the reaction of 2-ferrocenylbenzoic acid and 2-amino-6-methylpyridine, crystallises with two dissimilar molecules (A; Fig. 1) and (B; Fig. 2) in the asymmetric unit of space group *R3c*. Molecules aggregate into dimers via cyclic, asymmetric N—H···N interactions with graph set R^2^_2_(8), Fig. 3, further augmented via intramolecular C—H···O=C and interdimer C—H···π(arene) interactions, Table 1. Dimers are linked into chains via weak C—H···O contacts, Table 1.

The individual molecules do not exhibit any disorder and no rotational disorder was detected in the methyl-H atoms. Although the five carbon atoms in the unsubstituted η-C_5_H_5_ ring of molecule B show increased librational motion, there is no evidence for disorder in this group. The molecules of (A) and (B) are distinctly different and differ principally due to the orientation of the picoline amide group in relation to the 2-ferrocenylbenzene moiety. Differences between molecules (A) and (B) can be highlighted by analysis and comparison of the geometric data. The O1—C1—C32—C31 torsion angle is -123.4 (5)° (syn) in (A) and 51.5 (7)° (anti) in (B), whereas, the Fe1···O1A distance is 5.364 (4) Å as compared to Fe2···O1B with 4.841 (4) Å; the Fe1···N6A, Fe2···N6B distances are 6.544 (4) and 7.334 (4) Å, respectively. The interplanar angles between the seven atom picoline group [C2–C7] and the substituted η-C_5_H_4_ ring are 60.71 (17) ° (A) and 39.73 (19) ° (B). In fact, the superposition of molecule (A) and molecule (B) via a best atom fit in PLATON (Spek, 2009) produces a superposition whereby the aromatic chain has a reasonable fit but the ferrocene moieties are rotated by *ca*. 90° with respect to one another (Fig. 4).

### Experimental

Compound (I) was synthesized via standard condensation procedures and similar to the related syntheses reported by us (Gallagher *et al.*, 2008, 2009). Separation of the 1:1 and 2:1 derivatives was undertaken by using flash chromatography using CHCl_3_:ethyl acetate. Typical organic workup and washing gave the product (I) in modest yield of 30-40% as a 1:1 component of the mixture. Crystals suitable for X-ray diffraction were grown from CHCl_3_ as colourless blocks over a period of 1-2 weeks. The compounds gave clean ^1^H and ^13^C NMR spectra in CDCl_3_ and infrared spectra (in CHCl_3_ solution, and as KBr disks).

### Refinement

H atoms attached to C atoms were treated as riding using the SHELXL97 (Sheldrick, 2008) defaults at 150 (1) K with C—H = 0.95 Å (aromatic) and 0.98 Å (CH_3_) and U_iso_(H) = 1.2U_eq_(C) (aromatic) and 1.5U_eq_(CH_3_).

### Crystal data

**Table:**

[Fe(C_5_H_5_)(C_18_H_15_N_2_O)]	? #Insert any comments here.
*M**_r_* = 396.26	*D*_x_ = 1.422 Mg m^−^^3^
Trigonal, *R*3*c*	Mo *K*α radiation, λ = 0.71073 Å
Hall symbol: R 3 -2"c	Cell parameters from 7571 reflections
*a* = 22.8234 (9) Å	θ = 2.5–27.5°
*c* = 36.9342 (15) Å	µ = 0.83 mm^−^^1^
*V* = 16661.7 (11) Å^3^	*T* = 150 K
*Z* = 36	Block, red
*F*(000) = 7416	0.30 × 0.25 × 0.20 mm

### Data collection

**Table:**

Nonius KappaCCD diffractometer	6243 independent reflections
Radiation source: fine-focus sealed X-ray tube	4606 reflections with *I* > 2σ(*I*)
graphite	*R*_int_ = 0.085
φ, ω scans with κ offsets	θ_max_ = 25.1°, θ_min_ = 2.8°
Absorption correction: multi-scan (*SORTAV*; Blessing, 1995)	*h* = −26→27
*T*_min_ = 0.788, *T*_max_ = 0.851	*k* = −22→27
16925 measured reflections	*l* = −44→44

### Refinement

**Table:**

Refinement on *F*^2^	Hydrogen site location: inferred from neighbouring sites
Least-squares matrix: full	H-atom parameters constrained
*R*[*F*^2^ > 2σ(*F*^2^)] = 0.047	*w* = 1/[σ^2^(*F*_o_^2^) + (0.0293*P*)^2^] where *P* = (*F*_o_^2^ + 2*F*_c_^2^)/3
*wR*(*F*^2^) = 0.099	(Δ/σ)_max_ = 0.001
*S* = 1.01	Δρ_max_ = 0.34 e Å^−^^3^
6243 reflections	Δρ_min_ = −0.32 e Å^−^^3^
490 parameters	Extinction correction: *SHELXL97* (Sheldrick, 2008), Fc^*^=kFc[1+0.001xFc^2^λ^3^/sin(2θ)]^-1/4^
1 restraint	Extinction coefficient: 0.000083 (13)
Primary atom site location: structure-invariant direct methods	Absolute structure: Flack (1983), 2941 Friedel pairs
Secondary atom site location: difference Fourier map	Flack parameter: 0.016 (18)

### Special details

**Table:**

Experimental. ? #Insert any special details here.

### Fractional atomic coordinates and isotropic or equivalent isotropic displacement parameters (Å^2^)

**Table:**

	*x*	*y*	*z*	*U*_iso_*/*U*_eq_	
Fe1	0.50804 (4)	−0.14058 (4)	0.366994 (17)	0.03041 (18)	
O1A	0.57964 (17)	0.06331 (18)	0.46101 (8)	0.0400 (9)	
C1A	0.5487 (2)	0.0564 (2)	0.43285 (12)	0.0307 (12)	
N1A	0.5742 (2)	0.1010 (2)	0.40451 (10)	0.0329 (10)	
C2A	0.6361 (2)	0.1619 (2)	0.40334 (13)	0.0304 (11)	
C3A	0.6777 (3)	0.1920 (3)	0.43291 (14)	0.0402 (13)	
C4A	0.7357 (3)	0.2530 (3)	0.42786 (15)	0.0463 (14)	
C5A	0.7516 (3)	0.2829 (3)	0.39425 (16)	0.0479 (15)	
C6A	0.7084 (3)	0.2492 (3)	0.36536 (14)	0.0364 (12)	
N6A	0.6509 (2)	0.1895 (2)	0.36968 (10)	0.0325 (10)	
C7A	0.7260 (3)	0.2778 (3)	0.32778 (14)	0.0483 (15)	
C11A	0.5034 (2)	−0.0544 (2)	0.37597 (12)	0.0295 (11)	
C12A	0.5684 (2)	−0.0443 (3)	0.38589 (13)	0.0359 (12)	
C13A	0.5978 (2)	−0.0558 (3)	0.35475 (12)	0.0343 (12)	
C14A	0.5515 (3)	−0.0723 (2)	0.32600 (13)	0.0371 (13)	
C15A	0.4937 (3)	−0.0716 (2)	0.33915 (13)	0.0334 (11)	
C21A	0.4454 (3)	−0.2125 (3)	0.40302 (17)	0.0564 (17)	
C22A	0.5093 (3)	−0.2068 (3)	0.40364 (15)	0.0520 (16)	
C23A	0.5234 (3)	−0.2213 (3)	0.36932 (15)	0.0438 (14)	
C24A	0.4697 (3)	−0.2356 (3)	0.34657 (15)	0.0463 (15)	
C25A	0.4201 (3)	−0.2311 (3)	0.3679 (2)	0.0579 (17)	
C31A	0.4549 (2)	−0.0484 (2)	0.40025 (12)	0.0304 (11)	
C32A	0.4772 (2)	−0.0007 (2)	0.42779 (12)	0.0313 (12)	
C33A	0.4314 (3)	−0.0013 (3)	0.45290 (13)	0.0386 (13)	
C34A	0.3634 (3)	−0.0454 (3)	0.44892 (14)	0.0420 (14)	
C35A	0.3401 (3)	−0.0903 (3)	0.42023 (13)	0.0427 (14)	
C36A	0.3853 (2)	−0.0928 (3)	0.39645 (13)	0.0351 (12)	
Fe2	0.75675 (4)	0.24247 (4)	0.20017 (2)	0.0447 (2)	
O1B	0.59914 (18)	0.03559 (17)	0.26183 (8)	0.0401 (9)	
C1B	0.6231 (3)	0.0788 (2)	0.28568 (12)	0.0337 (12)	
N1B	0.5843 (2)	0.0959 (2)	0.30677 (10)	0.0327 (10)	
C2B	0.5134 (3)	0.0664 (2)	0.30552 (13)	0.0325 (12)	
C3B	0.4736 (3)	0.0285 (3)	0.27629 (13)	0.0395 (13)	
C4B	0.4048 (3)	0.0016 (3)	0.27882 (13)	0.0435 (14)	
C5B	0.3770 (3)	0.0133 (3)	0.30940 (13)	0.0437 (14)	
C6B	0.4198 (3)	0.0534 (3)	0.33658 (13)	0.0370 (13)	
N6B	0.4872 (2)	0.0781 (2)	0.33541 (10)	0.0310 (10)	
C7B	0.3923 (3)	0.0691 (3)	0.36966 (13)	0.0444 (14)	
C11B	0.7250 (3)	0.1543 (3)	0.22829 (13)	0.0360 (12)	
C12B	0.7609 (3)	0.1553 (3)	0.19618 (14)	0.0408 (13)	
C13B	0.7272 (3)	0.1624 (3)	0.16588 (15)	0.0509 (15)	
C14B	0.6697 (3)	0.1647 (3)	0.17836 (14)	0.0488 (15)	
C15B	0.6687 (3)	0.1612 (3)	0.21681 (13)	0.0369 (13)	
C21B	0.8174 (3)	0.3209 (3)	0.23378 (19)	0.0610 (18)	
C22B	0.8532 (3)	0.3239 (3)	0.20214 (18)	0.0612 (18)	
C23B	0.7597 (3)	0.3296 (3)	0.1862 (3)	0.078 (2)	
C24B	0.8179 (3)	0.3297 (3)	0.1727 (2)	0.069 (2)	
C25B	0.7593 (4)	0.3239 (3)	0.2240 (3)	0.076 (2)	
C31B	0.7450 (3)	0.1491 (3)	0.26582 (13)	0.0366 (13)	
C32B	0.6971 (3)	0.1165 (3)	0.29373 (12)	0.0344 (12)	
C33B	0.7189 (3)	0.1147 (3)	0.32885 (14)	0.0396 (13)	
C34B	0.7865 (3)	0.1428 (3)	0.33625 (14)	0.0445 (15)	
C35B	0.8341 (3)	0.1738 (3)	0.30914 (15)	0.0493 (15)	
C36B	0.8138 (3)	0.1777 (3)	0.27450 (14)	0.0423 (13)	
H1A	0.5490	0.0901	0.3849	0.040*	
H3A	0.6665	0.1710	0.4560	0.048*	
H4A	0.7651	0.2747	0.4477	0.056*	
H5A	0.7912	0.3257	0.3908	0.058*	
H7A1	0.7411	0.2517	0.3134	0.072*	
H7A2	0.7623	0.3252	0.3290	0.072*	
H7A3	0.6860	0.2754	0.3164	0.072*	
H12A	0.5884	−0.0319	0.4092	0.043*	
H13A	0.6407	−0.0529	0.3535	0.041*	
H14A	0.5580	−0.0822	0.3019	0.045*	
H15A	0.4548	−0.0812	0.3253	0.040*	
H21A	0.4233	−0.2050	0.4228	0.068*	
H22A	0.5382	−0.1951	0.4241	0.062*	
H23A	0.5635	−0.2215	0.3625	0.053*	
H24A	0.4665	−0.2463	0.3215	0.056*	
H25A	0.3772	−0.2394	0.3596	0.069*	
H33A	0.4474	0.0290	0.4729	0.046*	
H34A	0.3323	−0.0450	0.4659	0.050*	
H35A	0.2929	−0.1194	0.4169	0.051*	
H36A	0.3691	−0.1250	0.3773	0.042*	
H1B	0.6060	0.1285	0.3226	0.039*	
H3B	0.4935	0.0216	0.2553	0.047*	
H4B	0.3763	−0.0252	0.2596	0.052*	
H5B	0.3294	−0.0058	0.3116	0.052*	
H7B1	0.4103	0.0588	0.3913	0.067*	
H7B2	0.3428	0.0416	0.3696	0.067*	
H7B3	0.4058	0.1171	0.3697	0.067*	
H12B	0.8007	0.1519	0.1954	0.049*	
H13B	0.7407	0.1651	0.1413	0.061*	
H14B	0.6374	0.1680	0.1636	0.059*	
H15B	0.6364	0.1631	0.2322	0.044*	
H21B	0.8304	0.3174	0.2578	0.073*	
H22B	0.8939	0.3223	0.2010	0.073*	
H23B	0.7266	0.3328	0.1721	0.093*	
H24B	0.8307	0.3332	0.1480	0.083*	
H25B	0.7261	0.3224	0.2401	0.092*	
H33B	0.6866	0.0939	0.3477	0.047*	
H34B	0.8008	0.1410	0.3602	0.053*	
H35B	0.8809	0.1924	0.3144	0.059*	
H36B	0.8469	0.2001	0.2562	0.051*	

### Atomic displacement parameters (Å^2^)

**Table:**

	*U*^11^	*U*^22^	*U*^33^	*U*^12^	*U*^13^	*U*^23^
Fe1	0.0272 (4)	0.0289 (4)	0.0357 (3)	0.0144 (4)	0.0013 (3)	−0.0019 (3)
O1A	0.038 (2)	0.044 (2)	0.0380 (19)	0.0196 (19)	−0.0092 (17)	−0.0015 (17)
C1A	0.032 (3)	0.028 (3)	0.037 (3)	0.019 (3)	0.005 (2)	−0.005 (2)
N1A	0.032 (3)	0.028 (2)	0.033 (2)	0.011 (2)	−0.0009 (19)	−0.0044 (19)
C2A	0.029 (3)	0.023 (3)	0.039 (3)	0.012 (2)	−0.003 (2)	−0.007 (2)
C3A	0.037 (3)	0.035 (3)	0.047 (3)	0.017 (3)	−0.013 (3)	−0.013 (3)
C4A	0.037 (4)	0.038 (3)	0.055 (3)	0.013 (3)	−0.017 (3)	−0.012 (3)
C5A	0.034 (3)	0.030 (3)	0.073 (4)	0.011 (3)	−0.009 (3)	−0.008 (3)
C6A	0.036 (3)	0.028 (3)	0.049 (3)	0.019 (3)	0.004 (3)	−0.001 (2)
N6A	0.031 (3)	0.029 (2)	0.038 (2)	0.016 (2)	0.0023 (19)	−0.006 (2)
C7A	0.046 (4)	0.040 (4)	0.054 (3)	0.017 (3)	0.009 (3)	0.003 (3)
C11A	0.022 (3)	0.026 (3)	0.037 (3)	0.010 (2)	0.006 (2)	−0.004 (2)
C12A	0.025 (3)	0.032 (3)	0.045 (3)	0.010 (2)	−0.001 (2)	−0.013 (2)
C13A	0.021 (3)	0.034 (3)	0.045 (3)	0.012 (2)	0.006 (2)	−0.007 (2)
C14A	0.041 (3)	0.035 (3)	0.038 (3)	0.021 (3)	0.012 (3)	0.001 (2)
C15A	0.033 (3)	0.030 (3)	0.039 (3)	0.017 (3)	0.000 (2)	0.000 (2)
C21A	0.060 (5)	0.045 (4)	0.069 (4)	0.030 (4)	0.021 (4)	0.011 (3)
C22A	0.062 (4)	0.040 (4)	0.053 (4)	0.024 (3)	−0.005 (3)	0.006 (3)
C23A	0.040 (4)	0.043 (3)	0.056 (3)	0.026 (3)	0.008 (3)	0.009 (3)
C24A	0.055 (4)	0.030 (3)	0.050 (3)	0.018 (3)	−0.003 (3)	−0.005 (3)
C25A	0.032 (4)	0.028 (4)	0.111 (5)	0.013 (3)	−0.003 (4)	0.007 (3)
C31A	0.026 (3)	0.027 (3)	0.039 (3)	0.014 (2)	0.004 (2)	−0.003 (2)
C32A	0.031 (3)	0.030 (3)	0.039 (3)	0.019 (3)	0.003 (2)	−0.003 (2)
C33A	0.040 (3)	0.041 (3)	0.040 (3)	0.024 (3)	0.009 (3)	−0.007 (2)
C34A	0.045 (4)	0.038 (3)	0.047 (3)	0.023 (3)	0.016 (3)	0.000 (3)
C35A	0.025 (3)	0.050 (4)	0.054 (3)	0.020 (3)	0.009 (2)	−0.002 (3)
C36A	0.027 (3)	0.033 (3)	0.043 (3)	0.014 (3)	0.000 (2)	−0.011 (2)
Fe2	0.0383 (5)	0.0357 (5)	0.0649 (5)	0.0220 (4)	0.0165 (4)	0.0114 (4)
O1B	0.037 (2)	0.033 (2)	0.0414 (19)	0.0103 (18)	0.0025 (17)	−0.0091 (17)
C1B	0.043 (3)	0.028 (3)	0.031 (3)	0.018 (3)	0.008 (2)	0.002 (2)
N1B	0.030 (3)	0.029 (2)	0.033 (2)	0.009 (2)	−0.0003 (18)	−0.0051 (18)
C2B	0.035 (3)	0.025 (3)	0.037 (3)	0.015 (3)	0.002 (2)	0.002 (2)
C3B	0.044 (3)	0.037 (3)	0.034 (3)	0.018 (3)	−0.002 (2)	−0.003 (2)
C4B	0.039 (4)	0.049 (4)	0.040 (3)	0.021 (3)	−0.011 (3)	−0.007 (3)
C5B	0.033 (3)	0.044 (4)	0.047 (3)	0.014 (3)	−0.007 (3)	−0.008 (3)
C6B	0.031 (3)	0.040 (3)	0.045 (3)	0.021 (3)	−0.002 (3)	0.000 (3)
N6B	0.036 (3)	0.032 (2)	0.029 (2)	0.019 (2)	0.0030 (18)	0.0023 (18)
C7B	0.045 (4)	0.055 (4)	0.042 (3)	0.031 (3)	0.003 (3)	−0.002 (3)
C11B	0.039 (3)	0.030 (3)	0.044 (3)	0.022 (3)	0.009 (2)	0.004 (2)
C12B	0.040 (3)	0.038 (3)	0.050 (3)	0.023 (3)	0.017 (3)	0.002 (3)
C13B	0.064 (4)	0.051 (4)	0.045 (3)	0.034 (4)	0.014 (3)	0.006 (3)
C14B	0.053 (4)	0.047 (4)	0.050 (3)	0.027 (3)	0.001 (3)	0.005 (3)
C15B	0.033 (3)	0.034 (3)	0.046 (3)	0.018 (3)	0.007 (2)	0.001 (2)
C21B	0.043 (4)	0.035 (4)	0.097 (5)	0.014 (3)	0.024 (4)	0.008 (3)
C22B	0.037 (4)	0.040 (4)	0.102 (5)	0.016 (3)	0.021 (4)	0.016 (4)
C23B	0.040 (4)	0.040 (4)	0.158 (8)	0.023 (4)	0.023 (5)	0.039 (5)
C24B	0.049 (4)	0.044 (4)	0.108 (5)	0.018 (4)	0.024 (4)	0.029 (4)
C25B	0.057 (5)	0.023 (4)	0.138 (7)	0.011 (3)	0.044 (5)	0.009 (4)
C31B	0.038 (3)	0.028 (3)	0.047 (3)	0.019 (3)	0.006 (2)	−0.003 (2)
C32B	0.031 (3)	0.029 (3)	0.045 (3)	0.016 (3)	0.002 (2)	−0.001 (2)
C33B	0.046 (4)	0.029 (3)	0.042 (3)	0.018 (3)	0.004 (3)	0.002 (2)
C34B	0.055 (4)	0.042 (4)	0.042 (3)	0.029 (3)	−0.007 (3)	−0.006 (3)
C35B	0.043 (4)	0.049 (4)	0.062 (4)	0.027 (3)	−0.010 (3)	−0.014 (3)
C36B	0.042 (4)	0.033 (3)	0.055 (3)	0.021 (3)	0.010 (3)	−0.002 (3)

### Geometric parameters (Å, °)

**Table:**

Fe1—C11A	2.048 (5)	Fe2—C11B	2.048 (5)
Fe1—C12A	2.047 (5)	Fe2—C12B	2.044 (5)
Fe1—C13A	2.045 (5)	Fe2—C13B	2.041 (6)
Fe1—C14A	2.040 (5)	Fe2—C14B	2.054 (6)
Fe1—C15A	2.039 (5)	Fe2—C15B	2.031 (5)
Fe1—C21A	2.040 (6)	Fe2—C21B	2.045 (7)
Fe1—C22A	2.040 (5)	Fe2—C22B	2.053 (6)
Fe1—C23A	2.043 (5)	Fe2—C23B	2.023 (6)
Fe1—C24A	2.035 (5)	Fe2—C24B	2.041 (6)
Fe1—C25A	2.037 (6)	Fe2—C25B	2.031 (7)
O1A—C1A	1.222 (5)	O1B—C1B	1.229 (5)
C1A—N1A	1.370 (6)	C1B—N1B	1.373 (6)
C1A—C32A	1.506 (7)	C1B—C32B	1.493 (7)
N1A—C2A	1.402 (6)	N1B—C2B	1.409 (6)
N1A—H1A	0.8800	N1B—H1B	0.8800
C2A—N6A	1.357 (6)	C2B—N6B	1.345 (6)
C2A—C3A	1.384 (6)	C2B—C3B	1.398 (7)
C3A—C4A	1.372 (7)	C3B—C4B	1.373 (7)
C3A—H3A	0.9500	C3B—H3B	0.9500
C4A—C5A	1.375 (7)	C4B—C5B	1.386 (7)
C4A—H4A	0.9500	C4B—H4B	0.9500
C5A—C6A	1.394 (7)	C5B—C6B	1.381 (7)
C5A—H5A	0.9500	C5B—H5B	0.9500
C6A—N6A	1.349 (6)	C6B—N6B	1.348 (6)
C6A—C7A	1.501 (7)	C6B—C7B	1.496 (6)
C7A—H7A1	0.9800	C7B—H7B1	0.9800
C7A—H7A2	0.9800	C7B—H7B2	0.9800
C7A—H7A3	0.9800	C7B—H7B3	0.9800
C11A—C15A	1.402 (6)	C11B—C15B	1.434 (7)
C11A—C12A	1.430 (7)	C11B—C12B	1.435 (7)
C11A—C31A	1.483 (6)	C11B—C31B	1.483 (7)
C12A—C13A	1.420 (6)	C12B—C13B	1.413 (8)
C12A—H12A	0.9500	C12B—H12B	0.9500
C13A—C14A	1.410 (7)	C13B—C14B	1.415 (8)
C13A—H13A	0.9500	C13B—H13B	0.9500
C14A—C15A	1.412 (7)	C14B—C15B	1.422 (7)
C14A—H14A	0.9500	C14B—H14B	0.9500
C15A—H15A	0.9500	C15B—H15B	0.9500
C21A—C25A	1.397 (8)	C21B—C22B	1.407 (8)
C21A—C22A	1.400 (8)	C21B—C25B	1.409 (9)
C21A—H21A	0.9500	C21B—H21B	0.9500
C22A—C23A	1.388 (7)	C22B—C24B	1.399 (9)
C22A—H22A	0.9500	C22B—H22B	0.9500
C23A—C24A	1.385 (8)	C23B—C25B	1.403 (10)
C23A—H23A	0.9500	C23B—C24B	1.417 (9)
C24A—C25A	1.423 (8)	C23B—H23B	0.9500
C24A—H24A	0.9500	C24B—H24B	0.9500
C25A—H25A	0.9500	C25B—H25B	0.9500
C31A—C32A	1.387 (6)	C31B—C36B	1.403 (7)
C31A—C36A	1.400 (7)	C31B—C32B	1.413 (7)
C32A—C33A	1.393 (6)	C32B—C33B	1.397 (6)
C33A—C34A	1.372 (7)	C33B—C34B	1.371 (8)
C33A—H33A	0.9500	C33B—H33B	0.9500
C34A—C35A	1.382 (7)	C34B—C35B	1.384 (7)
C34A—H34A	0.9500	C34B—H34B	0.9500
C35A—C36A	1.378 (6)	C35B—C36B	1.379 (7)
C35A—H35A	0.9500	C35B—H35B	0.9500
C36A—H36A	0.9500	C36B—H36B	0.9500
			
C24A—Fe1—C25A	40.9 (2)	C23B—Fe2—C25B	40.5 (3)
C24A—Fe1—C15A	119.1 (2)	C23B—Fe2—C15B	120.4 (2)
C25A—Fe1—C15A	109.5 (2)	C25B—Fe2—C15B	105.5 (2)
C24A—Fe1—C14A	109.0 (2)	C23B—Fe2—C24B	40.8 (2)
C25A—Fe1—C14A	130.0 (3)	C25B—Fe2—C24B	68.3 (3)
C15A—Fe1—C14A	40.51 (19)	C15B—Fe2—C24B	157.3 (3)
C24A—Fe1—C21A	68.3 (3)	C23B—Fe2—C13B	123.8 (3)
C25A—Fe1—C21A	40.1 (2)	C25B—Fe2—C13B	159.1 (3)
C15A—Fe1—C21A	129.1 (2)	C15B—Fe2—C13B	68.7 (2)
C14A—Fe1—C21A	167.5 (3)	C24B—Fe2—C13B	108.9 (3)
C24A—Fe1—C22A	67.3 (2)	C23B—Fe2—C12B	160.7 (3)
C25A—Fe1—C22A	67.0 (3)	C25B—Fe2—C12B	158.1 (3)
C15A—Fe1—C22A	166.9 (2)	C15B—Fe2—C12B	68.9 (2)
C14A—Fe1—C22A	151.3 (2)	C24B—Fe2—C12B	124.9 (3)
C21A—Fe1—C22A	40.1 (2)	C13B—Fe2—C12B	40.5 (2)
C24A—Fe1—C23A	39.7 (2)	C23B—Fe2—C21B	67.6 (3)
C25A—Fe1—C23A	67.1 (2)	C25B—Fe2—C21B	40.4 (3)
C15A—Fe1—C23A	152.1 (2)	C15B—Fe2—C21B	122.9 (2)
C14A—Fe1—C23A	118.9 (2)	C24B—Fe2—C21B	67.3 (3)
C21A—Fe1—C23A	67.3 (2)	C13B—Fe2—C21B	159.4 (3)
C22A—Fe1—C23A	39.7 (2)	C12B—Fe2—C21B	123.7 (3)
C24A—Fe1—C13A	128.3 (2)	C23B—Fe2—C11B	156.5 (3)
C25A—Fe1—C13A	167.4 (2)	C25B—Fe2—C11B	120.9 (3)
C15A—Fe1—C13A	68.2 (2)	C15B—Fe2—C11B	41.15 (19)
C14A—Fe1—C13A	40.40 (19)	C24B—Fe2—C11B	160.8 (2)
C21A—Fe1—C13A	150.8 (2)	C13B—Fe2—C11B	68.8 (2)
C22A—Fe1—C13A	118.0 (2)	C12B—Fe2—C11B	41.07 (19)
C23A—Fe1—C13A	108.7 (2)	C21B—Fe2—C11B	107.7 (2)
C24A—Fe1—C12A	166.2 (2)	C23B—Fe2—C22B	68.0 (3)
C25A—Fe1—C12A	151.2 (2)	C25B—Fe2—C22B	68.2 (3)
C15A—Fe1—C12A	67.9 (2)	C15B—Fe2—C22B	159.9 (2)
C14A—Fe1—C12A	67.9 (2)	C24B—Fe2—C22B	40.0 (2)
C21A—Fe1—C12A	117.8 (2)	C13B—Fe2—C22B	123.9 (3)
C22A—Fe1—C12A	108.6 (2)	C12B—Fe2—C22B	109.5 (2)
C23A—Fe1—C12A	128.9 (2)	C21B—Fe2—C22B	40.2 (2)
C13A—Fe1—C12A	40.62 (18)	C11B—Fe2—C22B	124.4 (3)
C24A—Fe1—C11A	151.9 (2)	C23B—Fe2—C14B	107.0 (3)
C25A—Fe1—C11A	118.3 (2)	C25B—Fe2—C14B	122.3 (3)
C15A—Fe1—C11A	40.12 (18)	C15B—Fe2—C14B	40.73 (19)
C14A—Fe1—C11A	67.99 (19)	C24B—Fe2—C14B	122.8 (3)
C21A—Fe1—C11A	108.3 (2)	C13B—Fe2—C14B	40.4 (2)
C22A—Fe1—C11A	129.1 (2)	C12B—Fe2—C14B	68.1 (2)
C23A—Fe1—C11A	166.9 (2)	C21B—Fe2—C14B	159.0 (2)
C13A—Fe1—C11A	68.60 (19)	C11B—Fe2—C14B	68.6 (2)
C12A—Fe1—C11A	40.87 (19)	C22B—Fe2—C14B	158.8 (2)
O1A—C1A—N1A	123.4 (5)	O1B—C1B—N1B	122.9 (5)
O1A—C1A—C32A	121.5 (4)	O1B—C1B—C32B	121.8 (4)
N1A—C1A—C32A	115.0 (4)	N1B—C1B—C32B	115.3 (4)
C1A—N1A—C2A	127.1 (4)	C1B—N1B—C2B	126.5 (4)
C1A—N1A—H1A	116.4	C1B—N1B—H1B	116.7
C2A—N1A—H1A	116.4	C2B—N1B—H1B	116.7
N6A—C2A—C3A	122.9 (5)	N6B—C2B—C3B	122.7 (5)
N6A—C2A—N1A	112.4 (4)	N6B—C2B—N1B	113.4 (4)
C3A—C2A—N1A	124.7 (4)	C3B—C2B—N1B	123.9 (4)
C4A—C3A—C2A	118.1 (5)	C4B—C3B—C2B	117.9 (5)
C4A—C3A—H3A	121.0	C4B—C3B—H3B	121.0
C2A—C3A—H3A	121.0	C2B—C3B—H3B	121.0
C3A—C4A—C5A	120.6 (5)	C3B—C4B—C5B	120.1 (5)
C3A—C4A—H4A	119.7	C3B—C4B—H4B	120.0
C5A—C4A—H4A	119.7	C5B—C4B—H4B	120.0
C4A—C5A—C6A	118.6 (5)	C6B—C5B—C4B	118.6 (5)
C4A—C5A—H5A	120.7	C6B—C5B—H5B	120.7
C6A—C5A—H5A	120.7	C4B—C5B—H5B	120.7
N6A—C6A—C5A	122.0 (5)	N6B—C6B—C5B	122.4 (5)
N6A—C6A—C7A	117.6 (5)	N6B—C6B—C7B	117.0 (4)
C5A—C6A—C7A	120.4 (5)	C5B—C6B—C7B	120.6 (5)
C6A—N6A—C2A	117.9 (4)	C2B—N6B—C6B	118.1 (4)
C6A—C7A—H7A1	109.5	C6B—C7B—H7B1	109.5
C6A—C7A—H7A2	109.5	C6B—C7B—H7B2	109.5
H7A1—C7A—H7A2	109.5	H7B1—C7B—H7B2	109.5
C6A—C7A—H7A3	109.5	C6B—C7B—H7B3	109.5
H7A1—C7A—H7A3	109.5	H7B1—C7B—H7B3	109.5
H7A2—C7A—H7A3	109.5	H7B2—C7B—H7B3	109.5
C15A—C11A—C12A	107.4 (4)	C15B—C11B—C12B	106.8 (4)
C15A—C11A—C31A	126.3 (5)	C15B—C11B—C31B	127.9 (4)
C12A—C11A—C31A	126.3 (4)	C12B—C11B—C31B	125.2 (5)
C15A—C11A—Fe1	69.6 (3)	C15B—C11B—Fe2	68.8 (3)
C12A—C11A—Fe1	69.5 (3)	C12B—C11B—Fe2	69.3 (3)
C31A—C11A—Fe1	125.9 (3)	C31B—C11B—Fe2	125.5 (4)
C13A—C12A—C11A	108.1 (4)	C13B—C12B—C11B	108.4 (5)
C13A—C12A—Fe1	69.6 (3)	C13B—C12B—Fe2	69.7 (3)
C11A—C12A—Fe1	69.6 (3)	C11B—C12B—Fe2	69.6 (3)
C13A—C12A—H12A	126.0	C13B—C12B—H12B	125.8
C11A—C12A—H12A	126.0	C11B—C12B—H12B	125.8
Fe1—C12A—H12A	126.4	Fe2—C12B—H12B	126.5
C14A—C13A—C12A	107.4 (4)	C12B—C13B—C14B	108.3 (5)
C14A—C13A—Fe1	69.6 (3)	C12B—C13B—Fe2	69.9 (3)
C12A—C13A—Fe1	69.8 (3)	C14B—C13B—Fe2	70.3 (3)
C14A—C13A—H13A	126.3	C12B—C13B—H13B	125.8
C12A—C13A—H13A	126.3	C14B—C13B—H13B	125.8
Fe1—C13A—H13A	125.9	Fe2—C13B—H13B	125.6
C13A—C14A—C15A	108.5 (4)	C13B—C14B—C15B	108.2 (5)
C13A—C14A—Fe1	70.0 (3)	C13B—C14B—Fe2	69.3 (3)
C15A—C14A—Fe1	69.7 (3)	C15B—C14B—Fe2	68.8 (3)
C13A—C14A—H14A	125.8	C13B—C14B—H14B	125.9
C15A—C14A—H14A	125.8	C15B—C14B—H14B	125.9
Fe1—C14A—H14A	126.1	Fe2—C14B—H14B	127.6
C11A—C15A—C14A	108.6 (5)	C14B—C15B—C11B	108.2 (5)
C11A—C15A—Fe1	70.3 (3)	C14B—C15B—Fe2	70.5 (3)
C14A—C15A—Fe1	69.8 (3)	C11B—C15B—Fe2	70.1 (3)
C11A—C15A—H15A	125.7	C14B—C15B—H15B	125.9
C14A—C15A—H15A	125.7	C11B—C15B—H15B	125.9
Fe1—C15A—H15A	125.8	Fe2—C15B—H15B	125.1
C25A—C21A—C22A	107.2 (5)	C22B—C21B—C25B	108.8 (7)
C25A—C21A—Fe1	69.9 (3)	C22B—C21B—Fe2	70.2 (4)
C22A—C21A—Fe1	70.0 (3)	C25B—C21B—Fe2	69.2 (4)
C25A—C21A—H21A	126.4	C22B—C21B—H21B	125.6
C22A—C21A—H21A	126.4	C25B—C21B—H21B	125.6
Fe1—C21A—H21A	125.4	Fe2—C21B—H21B	126.5
C23A—C22A—C21A	108.6 (5)	C24B—C22B—C21B	107.7 (6)
C23A—C22A—Fe1	70.2 (3)	C24B—C22B—Fe2	69.6 (4)
C21A—C22A—Fe1	69.9 (3)	C21B—C22B—Fe2	69.6 (4)
C23A—C22A—H22A	125.7	C24B—C22B—H22B	126.2
C21A—C22A—H22A	125.7	C21B—C22B—H22B	126.2
Fe1—C22A—H22A	125.7	Fe2—C22B—H22B	126.2
C24A—C23A—C22A	109.1 (5)	C25B—C23B—C24B	108.2 (7)
C24A—C23A—Fe1	69.8 (3)	C25B—C23B—Fe2	70.1 (4)
C22A—C23A—Fe1	70.0 (3)	C24B—C23B—Fe2	70.3 (4)
C24A—C23A—H23A	125.5	C25B—C23B—H23B	125.9
C22A—C23A—H23A	125.5	C24B—C23B—H23B	125.9
Fe1—C23A—H23A	126.3	Fe2—C23B—H23B	125.4
C23A—C24A—C25A	106.8 (5)	C22B—C24B—C23B	108.0 (7)
C23A—C24A—Fe1	70.5 (3)	C22B—C24B—Fe2	70.5 (4)
C25A—C24A—Fe1	69.6 (3)	C23B—C24B—Fe2	68.9 (4)
C23A—C24A—H24A	126.6	C22B—C24B—H24B	126.0
C25A—C24A—H24A	126.6	C23B—C24B—H24B	126.0
Fe1—C24A—H24A	124.9	Fe2—C24B—H24B	126.2
C21A—C25A—C24A	108.3 (6)	C23B—C25B—C21B	107.3 (6)
C21A—C25A—Fe1	70.1 (4)	C23B—C25B—Fe2	69.4 (4)
C24A—C25A—Fe1	69.5 (3)	C21B—C25B—Fe2	70.3 (4)
C21A—C25A—H25A	125.8	C23B—C25B—H25B	126.4
C24A—C25A—H25A	125.8	C21B—C25B—H25B	126.4
Fe1—C25A—H25A	126.2	Fe2—C25B—H25B	125.5
C32A—C31A—C36A	118.6 (4)	C36B—C31B—C32B	118.0 (5)
C32A—C31A—C11A	121.2 (4)	C36B—C31B—C11B	119.6 (5)
C36A—C31A—C11A	120.1 (4)	C32B—C31B—C11B	122.4 (5)
C31A—C32A—C33A	120.1 (5)	C33B—C32B—C31B	120.0 (5)
C31A—C32A—C1A	125.7 (4)	C33B—C32B—C1B	119.0 (4)
C33A—C32A—C1A	114.1 (4)	C31B—C32B—C1B	120.8 (4)
C34A—C33A—C32A	120.3 (5)	C34B—C33B—C32B	120.3 (5)
C34A—C33A—H33A	119.8	C34B—C33B—H33B	119.8
C32A—C33A—H33A	119.8	C32B—C33B—H33B	119.8
C33A—C34A—C35A	120.0 (5)	C33B—C34B—C35B	120.5 (5)
C33A—C34A—H34A	120.0	C33B—C34B—H34B	119.7
C35A—C34A—H34A	120.0	C35B—C34B—H34B	119.7
C36A—C35A—C34A	120.1 (5)	C36B—C35B—C34B	120.0 (5)
C36A—C35A—H35A	120.0	C36B—C35B—H35B	120.0
C34A—C35A—H35A	120.0	C34B—C35B—H35B	120.0
C35A—C36A—C31A	120.6 (5)	C35B—C36B—C31B	121.1 (5)
C35A—C36A—H36A	119.7	C35B—C36B—H36B	119.4
C31A—C36A—H36A	119.7	C31B—C36B—H36B	119.4
			
O1A—C1A—N1A—C2A	−3.8 (7)	O1B—C1B—N1B—C2B	2.2 (8)
C32A—C1A—N1A—C2A	173.0 (4)	C32B—C1B—N1B—C2B	−177.6 (4)
C1A—N1A—C2A—N6A	171.1 (4)	C1B—N1B—C2B—N6B	161.5 (4)
C1A—N1A—C2A—C3A	−9.6 (8)	C1B—N1B—C2B—C3B	−18.4 (8)
N6A—C2A—C3A—C4A	1.4 (8)	N6B—C2B—C3B—C4B	−1.0 (8)
N1A—C2A—C3A—C4A	−177.8 (5)	N1B—C2B—C3B—C4B	178.9 (5)
C2A—C3A—C4A—C5A	−0.1 (8)	C2B—C3B—C4B—C5B	1.2 (8)
C3A—C4A—C5A—C6A	−1.5 (8)	C3B—C4B—C5B—C6B	1.2 (8)
C4A—C5A—C6A—N6A	2.0 (8)	C4B—C5B—C6B—N6B	−4.0 (8)
C4A—C5A—C6A—C7A	−176.2 (5)	C4B—C5B—C6B—C7B	178.4 (5)
C5A—C6A—N6A—C2A	−0.8 (7)	C3B—C2B—N6B—C6B	−1.6 (7)
C7A—C6A—N6A—C2A	177.4 (4)	N1B—C2B—N6B—C6B	178.4 (4)
C3A—C2A—N6A—C6A	−0.9 (7)	C5B—C6B—N6B—C2B	4.2 (7)
N1A—C2A—N6A—C6A	178.4 (4)	C7B—C6B—N6B—C2B	−178.1 (4)
C24A—Fe1—C11A—C15A	−51.7 (5)	C23B—Fe2—C11B—C15B	45.6 (8)
C25A—Fe1—C11A—C15A	−87.2 (4)	C25B—Fe2—C11B—C15B	77.8 (4)
C14A—Fe1—C11A—C15A	37.5 (3)	C24B—Fe2—C11B—C15B	−168.3 (8)
C21A—Fe1—C11A—C15A	−129.7 (3)	C13B—Fe2—C11B—C15B	−81.5 (3)
C22A—Fe1—C11A—C15A	−169.5 (3)	C12B—Fe2—C11B—C15B	−118.6 (4)
C23A—Fe1—C11A—C15A	161.8 (9)	C21B—Fe2—C11B—C15B	120.1 (3)
C13A—Fe1—C11A—C15A	81.2 (3)	C22B—Fe2—C11B—C15B	161.2 (3)
C12A—Fe1—C11A—C15A	118.7 (4)	C14B—Fe2—C11B—C15B	−37.9 (3)
C24A—Fe1—C11A—C12A	−170.4 (4)	C23B—Fe2—C11B—C12B	164.2 (7)
C25A—Fe1—C11A—C12A	154.1 (3)	C25B—Fe2—C11B—C12B	−163.6 (4)
C15A—Fe1—C11A—C12A	−118.7 (4)	C15B—Fe2—C11B—C12B	118.6 (4)
C14A—Fe1—C11A—C12A	−81.2 (3)	C24B—Fe2—C11B—C12B	−49.6 (10)
C21A—Fe1—C11A—C12A	111.6 (3)	C13B—Fe2—C11B—C12B	37.2 (3)
C22A—Fe1—C11A—C12A	71.8 (4)	C21B—Fe2—C11B—C12B	−121.3 (3)
C23A—Fe1—C11A—C12A	43.1 (10)	C22B—Fe2—C11B—C12B	−80.2 (4)
C13A—Fe1—C11A—C12A	−37.5 (3)	C14B—Fe2—C11B—C12B	80.7 (3)
C24A—Fe1—C11A—C31A	69.0 (6)	C23B—Fe2—C11B—C31B	−76.6 (9)
C25A—Fe1—C11A—C31A	33.5 (5)	C25B—Fe2—C11B—C31B	−44.3 (6)
C15A—Fe1—C11A—C31A	120.7 (6)	C15B—Fe2—C11B—C31B	−122.2 (5)
C14A—Fe1—C11A—C31A	158.2 (5)	C24B—Fe2—C11B—C31B	69.6 (10)
C21A—Fe1—C11A—C31A	−9.0 (5)	C13B—Fe2—C11B—C31B	156.4 (5)
C22A—Fe1—C11A—C31A	−48.8 (5)	C12B—Fe2—C11B—C31B	119.2 (6)
C23A—Fe1—C11A—C31A	−77.5 (11)	C21B—Fe2—C11B—C31B	−2.1 (5)
C13A—Fe1—C11A—C31A	−158.1 (5)	C22B—Fe2—C11B—C31B	39.0 (5)
C12A—Fe1—C11A—C31A	−120.6 (5)	C14B—Fe2—C11B—C31B	−160.1 (5)
C15A—C11A—C12A—C13A	−0.3 (5)	C15B—C11B—C12B—C13B	−0.3 (6)
C31A—C11A—C12A—C13A	179.3 (5)	C31B—C11B—C12B—C13B	−178.6 (5)
Fe1—C11A—C12A—C13A	59.2 (3)	Fe2—C11B—C12B—C13B	−59.0 (4)
C15A—C11A—C12A—Fe1	−59.5 (3)	C15B—C11B—C12B—Fe2	58.8 (4)
C31A—C11A—C12A—Fe1	120.1 (5)	C31B—C11B—C12B—Fe2	−119.6 (5)
C24A—Fe1—C12A—C13A	41.4 (11)	C23B—Fe2—C12B—C13B	−41.0 (9)
C25A—Fe1—C12A—C13A	−172.5 (5)	C25B—Fe2—C12B—C13B	160.4 (6)
C15A—Fe1—C12A—C13A	−81.8 (3)	C15B—Fe2—C12B—C13B	81.6 (4)
C14A—Fe1—C12A—C13A	−37.9 (3)	C24B—Fe2—C12B—C13B	−78.0 (5)
C21A—Fe1—C12A—C13A	154.4 (3)	C21B—Fe2—C12B—C13B	−162.2 (4)
C22A—Fe1—C12A—C13A	111.7 (3)	C11B—Fe2—C12B—C13B	119.8 (5)
C23A—Fe1—C12A—C13A	72.1 (4)	C22B—Fe2—C12B—C13B	−119.8 (4)
C11A—Fe1—C12A—C13A	−119.4 (4)	C14B—Fe2—C12B—C13B	37.6 (3)
C24A—Fe1—C12A—C11A	160.8 (9)	C23B—Fe2—C12B—C11B	−160.8 (7)
C25A—Fe1—C12A—C11A	−53.1 (6)	C25B—Fe2—C12B—C11B	40.6 (8)
C15A—Fe1—C12A—C11A	37.6 (3)	C15B—Fe2—C12B—C11B	−38.3 (3)
C14A—Fe1—C12A—C11A	81.5 (3)	C24B—Fe2—C12B—C11B	162.2 (4)
C21A—Fe1—C12A—C11A	−86.2 (3)	C13B—Fe2—C12B—C11B	−119.8 (5)
C22A—Fe1—C12A—C11A	−128.9 (3)	C21B—Fe2—C12B—C11B	78.0 (4)
C23A—Fe1—C12A—C11A	−168.5 (3)	C22B—Fe2—C12B—C11B	120.4 (3)
C13A—Fe1—C12A—C11A	119.4 (4)	C14B—Fe2—C12B—C11B	−82.2 (3)
C11A—C12A—C13A—C14A	0.5 (6)	C11B—C12B—C13B—C14B	−0.9 (6)
Fe1—C12A—C13A—C14A	59.7 (4)	Fe2—C12B—C13B—C14B	−60.0 (4)
C11A—C12A—C13A—Fe1	−59.2 (3)	C11B—C12B—C13B—Fe2	59.0 (4)
C24A—Fe1—C13A—C14A	73.1 (4)	C23B—Fe2—C13B—C12B	164.9 (3)
C25A—Fe1—C13A—C14A	44.7 (12)	C25B—Fe2—C13B—C12B	−159.5 (7)
C15A—Fe1—C13A—C14A	−37.5 (3)	C15B—Fe2—C13B—C12B	−82.0 (3)
C21A—Fe1—C13A—C14A	−170.2 (4)	C24B—Fe2—C13B—C12B	122.0 (4)
C22A—Fe1—C13A—C14A	155.3 (3)	C21B—Fe2—C13B—C12B	46.3 (9)
C23A—Fe1—C13A—C14A	113.0 (3)	C11B—Fe2—C13B—C12B	−37.7 (3)
C12A—Fe1—C13A—C14A	−118.5 (4)	C22B—Fe2—C13B—C12B	80.3 (4)
C11A—Fe1—C13A—C14A	−80.7 (3)	C14B—Fe2—C13B—C12B	−119.2 (5)
C24A—Fe1—C13A—C12A	−168.4 (3)	C23B—Fe2—C13B—C14B	−75.9 (4)
C25A—Fe1—C13A—C12A	163.2 (10)	C25B—Fe2—C13B—C14B	−40.3 (9)
C15A—Fe1—C13A—C12A	81.0 (3)	C15B—Fe2—C13B—C14B	37.2 (3)
C14A—Fe1—C13A—C12A	118.5 (4)	C24B—Fe2—C13B—C14B	−118.8 (4)
C21A—Fe1—C13A—C12A	−51.7 (6)	C12B—Fe2—C13B—C14B	119.2 (5)
C22A—Fe1—C13A—C12A	−86.2 (4)	C21B—Fe2—C13B—C14B	165.5 (7)
C23A—Fe1—C13A—C12A	−128.5 (3)	C11B—Fe2—C13B—C14B	81.5 (3)
C11A—Fe1—C13A—C12A	37.8 (3)	C22B—Fe2—C13B—C14B	−160.5 (3)
C12A—C13A—C14A—C15A	−0.5 (6)	C12B—C13B—C14B—C15B	1.8 (7)
Fe1—C13A—C14A—C15A	59.3 (3)	Fe2—C13B—C14B—C15B	−57.9 (4)
C12A—C13A—C14A—Fe1	−59.8 (3)	C12B—C13B—C14B—Fe2	59.7 (4)
C24A—Fe1—C14A—C13A	−127.5 (3)	C23B—Fe2—C14B—C13B	122.5 (4)
C25A—Fe1—C14A—C13A	−168.5 (3)	C25B—Fe2—C14B—C13B	164.1 (4)
C15A—Fe1—C14A—C13A	119.6 (4)	C15B—Fe2—C14B—C13B	−120.3 (5)
C21A—Fe1—C14A—C13A	157.6 (10)	C24B—Fe2—C14B—C13B	80.7 (4)
C22A—Fe1—C14A—C13A	−50.3 (6)	C12B—Fe2—C14B—C13B	−37.7 (3)
C23A—Fe1—C14A—C13A	−85.2 (3)	C21B—Fe2—C14B—C13B	−165.8 (7)
C12A—Fe1—C14A—C13A	38.1 (3)	C11B—Fe2—C14B—C13B	−82.0 (3)
C11A—Fe1—C14A—C13A	82.4 (3)	C22B—Fe2—C14B—C13B	49.8 (8)
C24A—Fe1—C14A—C15A	112.9 (3)	C23B—Fe2—C14B—C15B	−117.2 (4)
C25A—Fe1—C14A—C15A	71.9 (4)	C25B—Fe2—C14B—C15B	−75.6 (4)
C21A—Fe1—C14A—C15A	38.0 (12)	C24B—Fe2—C14B—C15B	−159.0 (3)
C22A—Fe1—C14A—C15A	−169.9 (4)	C13B—Fe2—C14B—C15B	120.3 (5)
C23A—Fe1—C14A—C15A	155.2 (3)	C12B—Fe2—C14B—C15B	82.6 (3)
C13A—Fe1—C14A—C15A	−119.6 (4)	C21B—Fe2—C14B—C15B	−45.5 (8)
C12A—Fe1—C14A—C15A	−81.5 (3)	C11B—Fe2—C14B—C15B	38.3 (3)
C11A—Fe1—C14A—C15A	−37.2 (3)	C22B—Fe2—C14B—C15B	170.1 (6)
C12A—C11A—C15A—C14A	0.0 (5)	C13B—C14B—C15B—C11B	−2.0 (6)
C31A—C11A—C15A—C14A	−179.6 (4)	Fe2—C14B—C15B—C11B	−60.2 (4)
Fe1—C11A—C15A—C14A	−59.4 (3)	C13B—C14B—C15B—Fe2	58.2 (4)
C12A—C11A—C15A—Fe1	59.5 (3)	C12B—C11B—C15B—C14B	1.4 (6)
C31A—C11A—C15A—Fe1	−120.2 (5)	C31B—C11B—C15B—C14B	179.7 (5)
C13A—C14A—C15A—C11A	0.3 (6)	Fe2—C11B—C15B—C14B	60.5 (4)
Fe1—C14A—C15A—C11A	59.8 (3)	C12B—C11B—C15B—Fe2	−59.1 (4)
C13A—C14A—C15A—Fe1	−59.5 (4)	C31B—C11B—C15B—Fe2	119.2 (5)
C24A—Fe1—C15A—C11A	154.9 (3)	C23B—Fe2—C15B—C14B	80.6 (5)
C25A—Fe1—C15A—C11A	111.0 (3)	C25B—Fe2—C15B—C14B	121.8 (4)
C14A—Fe1—C15A—C11A	−119.6 (4)	C24B—Fe2—C15B—C14B	51.3 (8)
C21A—Fe1—C15A—C11A	70.3 (4)	C13B—Fe2—C15B—C14B	−36.9 (3)
C22A—Fe1—C15A—C11A	38.7 (11)	C12B—Fe2—C15B—C14B	−80.5 (3)
C23A—Fe1—C15A—C11A	−171.3 (4)	C21B—Fe2—C15B—C14B	162.2 (4)
C13A—Fe1—C15A—C11A	−82.2 (3)	C11B—Fe2—C15B—C14B	−118.7 (4)
C12A—Fe1—C15A—C11A	−38.3 (3)	C22B—Fe2—C15B—C14B	−169.6 (6)
C24A—Fe1—C15A—C14A	−85.5 (4)	C23B—Fe2—C15B—C11B	−160.7 (4)
C25A—Fe1—C15A—C14A	−129.4 (3)	C25B—Fe2—C15B—C11B	−119.5 (4)
C21A—Fe1—C15A—C14A	−170.1 (3)	C24B—Fe2—C15B—C11B	170.0 (7)
C22A—Fe1—C15A—C14A	158.3 (10)	C13B—Fe2—C15B—C11B	81.8 (3)
C23A—Fe1—C15A—C14A	−51.7 (6)	C12B—Fe2—C15B—C11B	38.2 (3)
C13A—Fe1—C15A—C14A	37.4 (3)	C21B—Fe2—C15B—C11B	−79.1 (4)
C12A—Fe1—C15A—C14A	81.3 (3)	C22B—Fe2—C15B—C11B	−50.9 (8)
C11A—Fe1—C15A—C14A	119.6 (4)	C14B—Fe2—C15B—C11B	118.7 (4)
C24A—Fe1—C21A—C25A	−37.8 (4)	C23B—Fe2—C21B—C22B	−81.9 (4)
C15A—Fe1—C21A—C25A	72.7 (4)	C25B—Fe2—C21B—C22B	−120.2 (6)
C14A—Fe1—C21A—C25A	41.7 (12)	C15B—Fe2—C21B—C22B	165.4 (4)
C22A—Fe1—C21A—C25A	−117.9 (5)	C24B—Fe2—C21B—C22B	−37.5 (4)
C23A—Fe1—C21A—C25A	−80.8 (4)	C13B—Fe2—C21B—C22B	45.9 (9)
C13A—Fe1—C21A—C25A	−168.8 (4)	C12B—Fe2—C21B—C22B	80.3 (4)
C12A—Fe1—C21A—C25A	155.9 (3)	C11B—Fe2—C21B—C22B	122.7 (4)
C11A—Fe1—C21A—C25A	112.4 (4)	C14B—Fe2—C21B—C22B	−160.9 (6)
C24A—Fe1—C21A—C22A	80.1 (4)	C23B—Fe2—C21B—C25B	38.3 (4)
C25A—Fe1—C21A—C22A	117.9 (5)	C15B—Fe2—C21B—C25B	−74.4 (5)
C15A—Fe1—C21A—C22A	−169.4 (3)	C24B—Fe2—C21B—C25B	82.6 (5)
C14A—Fe1—C21A—C22A	159.6 (10)	C13B—Fe2—C21B—C25B	166.1 (7)
C23A—Fe1—C21A—C22A	37.1 (3)	C12B—Fe2—C21B—C25B	−159.5 (4)
C13A—Fe1—C21A—C22A	−50.9 (6)	C11B—Fe2—C21B—C25B	−117.1 (4)
C12A—Fe1—C21A—C22A	−86.2 (4)	C22B—Fe2—C21B—C25B	120.2 (6)
C11A—Fe1—C21A—C22A	−129.6 (4)	C14B—Fe2—C21B—C25B	−40.7 (9)
C25A—C21A—C22A—C23A	0.4 (7)	C25B—C21B—C22B—C24B	0.8 (7)
Fe1—C21A—C22A—C23A	−59.8 (4)	Fe2—C21B—C22B—C24B	59.4 (5)
C25A—C21A—C22A—Fe1	60.2 (4)	C25B—C21B—C22B—Fe2	−58.6 (4)
C24A—Fe1—C22A—C23A	36.7 (3)	C23B—Fe2—C22B—C24B	−38.0 (4)
C25A—Fe1—C22A—C23A	81.3 (4)	C25B—Fe2—C22B—C24B	−81.8 (5)
C15A—Fe1—C22A—C23A	158.6 (9)	C15B—Fe2—C22B—C24B	−156.9 (6)
C14A—Fe1—C22A—C23A	−51.5 (6)	C13B—Fe2—C22B—C24B	78.8 (5)
C21A—Fe1—C22A—C23A	119.5 (5)	C12B—Fe2—C22B—C24B	121.5 (4)
C13A—Fe1—C22A—C23A	−85.9 (4)	C21B—Fe2—C22B—C24B	−118.9 (6)
C12A—Fe1—C22A—C23A	−129.2 (3)	C11B—Fe2—C22B—C24B	164.9 (4)
C11A—Fe1—C22A—C23A	−170.2 (3)	C14B—Fe2—C22B—C24B	42.1 (9)
C24A—Fe1—C22A—C21A	−82.7 (4)	C23B—Fe2—C22B—C21B	81.0 (5)
C25A—Fe1—C22A—C21A	−38.2 (4)	C25B—Fe2—C22B—C21B	37.2 (4)
C15A—Fe1—C22A—C21A	39.1 (12)	C15B—Fe2—C22B—C21B	−38.0 (9)
C14A—Fe1—C22A—C21A	−171.0 (4)	C24B—Fe2—C22B—C21B	118.9 (6)
C23A—Fe1—C22A—C21A	−119.5 (5)	C13B—Fe2—C22B—C21B	−162.3 (4)
C13A—Fe1—C22A—C21A	154.6 (4)	C12B—Fe2—C22B—C21B	−119.5 (4)
C12A—Fe1—C22A—C21A	111.4 (4)	C11B—Fe2—C22B—C21B	−76.2 (4)
C11A—Fe1—C22A—C21A	70.4 (4)	C14B—Fe2—C22B—C21B	161.1 (6)
C21A—C22A—C23A—C24A	0.6 (7)	C15B—Fe2—C23B—C25B	77.9 (5)
Fe1—C22A—C23A—C24A	−59.1 (4)	C24B—Fe2—C23B—C25B	−118.9 (7)
C21A—C22A—C23A—Fe1	59.6 (4)	C13B—Fe2—C23B—C25B	161.3 (4)
C25A—Fe1—C23A—C24A	39.2 (4)	C12B—Fe2—C23B—C25B	−167.9 (7)
C15A—Fe1—C23A—C24A	−49.5 (6)	C21B—Fe2—C23B—C25B	−38.2 (4)
C14A—Fe1—C23A—C24A	−85.2 (4)	C11B—Fe2—C23B—C25B	44.8 (9)
C21A—Fe1—C23A—C24A	82.8 (4)	C22B—Fe2—C23B—C25B	−81.7 (4)
C22A—Fe1—C23A—C24A	120.3 (5)	C14B—Fe2—C23B—C25B	120.2 (4)
C13A—Fe1—C23A—C24A	−128.1 (3)	C25B—Fe2—C23B—C24B	118.9 (7)
C12A—Fe1—C23A—C24A	−169.0 (3)	C15B—Fe2—C23B—C24B	−163.2 (4)
C11A—Fe1—C23A—C24A	156.0 (8)	C13B—Fe2—C23B—C24B	−79.8 (5)
C24A—Fe1—C23A—C22A	−120.3 (5)	C12B—Fe2—C23B—C24B	−49.0 (10)
C25A—Fe1—C23A—C22A	−81.1 (4)	C21B—Fe2—C23B—C24B	80.7 (5)
C15A—Fe1—C23A—C22A	−169.8 (5)	C11B—Fe2—C23B—C24B	163.8 (6)
C14A—Fe1—C23A—C22A	154.6 (4)	C22B—Fe2—C23B—C24B	37.2 (4)
C21A—Fe1—C23A—C22A	−37.5 (4)	C14B—Fe2—C23B—C24B	−120.9 (5)
C13A—Fe1—C23A—C22A	111.6 (4)	C21B—C22B—C24B—C23B	−0.6 (7)
C12A—Fe1—C23A—C22A	70.7 (4)	Fe2—C22B—C24B—C23B	58.9 (5)
C11A—Fe1—C23A—C22A	35.7 (11)	C21B—C22B—C24B—Fe2	−59.5 (4)
C22A—C23A—C24A—C25A	−1.3 (6)	C25B—C23B—C24B—C22B	0.2 (8)
Fe1—C23A—C24A—C25A	−60.5 (4)	Fe2—C23B—C24B—C22B	−59.8 (5)
C22A—C23A—C24A—Fe1	59.2 (4)	C25B—C23B—C24B—Fe2	60.0 (5)
C25A—Fe1—C24A—C23A	−117.3 (5)	C23B—Fe2—C24B—C22B	119.3 (7)
C15A—Fe1—C24A—C23A	156.0 (3)	C25B—Fe2—C24B—C22B	81.5 (5)
C14A—Fe1—C24A—C23A	112.8 (4)	C15B—Fe2—C24B—C22B	159.5 (6)
C21A—Fe1—C24A—C23A	−80.3 (4)	C13B—Fe2—C24B—C22B	−120.6 (4)
C22A—Fe1—C24A—C23A	−36.8 (3)	C12B—Fe2—C24B—C22B	−78.5 (5)
C13A—Fe1—C24A—C23A	71.8 (4)	C21B—Fe2—C24B—C22B	37.7 (4)
C12A—Fe1—C24A—C23A	38.4 (11)	C11B—Fe2—C24B—C22B	−40.8 (11)
C11A—Fe1—C24A—C23A	−168.7 (4)	C14B—Fe2—C24B—C22B	−163.2 (4)
C15A—Fe1—C24A—C25A	−86.7 (4)	C25B—Fe2—C24B—C23B	−37.7 (5)
C14A—Fe1—C24A—C25A	−129.9 (4)	C15B—Fe2—C24B—C23B	40.2 (9)
C21A—Fe1—C24A—C25A	37.1 (4)	C13B—Fe2—C24B—C23B	120.1 (5)
C22A—Fe1—C24A—C25A	80.6 (4)	C12B—Fe2—C24B—C23B	162.3 (5)
C23A—Fe1—C24A—C25A	117.3 (5)	C21B—Fe2—C24B—C23B	−81.6 (5)
C13A—Fe1—C24A—C25A	−170.9 (4)	C11B—Fe2—C24B—C23B	−160.1 (8)
C12A—Fe1—C24A—C25A	155.8 (9)	C22B—Fe2—C24B—C23B	−119.3 (7)
C11A—Fe1—C24A—C25A	−51.3 (6)	C14B—Fe2—C24B—C23B	77.5 (6)
C22A—C21A—C25A—C24A	−1.2 (7)	C24B—C23B—C25B—C21B	0.3 (8)
Fe1—C21A—C25A—C24A	59.1 (4)	Fe2—C23B—C25B—C21B	60.4 (4)
C22A—C21A—C25A—Fe1	−60.3 (4)	C24B—C23B—C25B—Fe2	−60.2 (5)
C23A—C24A—C25A—C21A	1.5 (7)	C22B—C21B—C25B—C23B	−0.6 (7)
Fe1—C24A—C25A—C21A	−59.5 (4)	Fe2—C21B—C25B—C23B	−59.9 (5)
C23A—C24A—C25A—Fe1	61.0 (4)	C22B—C21B—C25B—Fe2	59.2 (4)
C24A—Fe1—C25A—C21A	119.6 (5)	C15B—Fe2—C25B—C23B	−119.0 (4)
C15A—Fe1—C25A—C21A	−128.2 (4)	C24B—Fe2—C25B—C23B	38.0 (4)
C14A—Fe1—C25A—C21A	−169.2 (3)	C13B—Fe2—C25B—C23B	−48.2 (9)
C22A—Fe1—C25A—C21A	38.2 (4)	C12B—Fe2—C25B—C23B	169.3 (6)
C23A—Fe1—C25A—C21A	81.5 (4)	C21B—Fe2—C25B—C23B	118.1 (6)
C13A—Fe1—C25A—C21A	154.3 (10)	C11B—Fe2—C25B—C23B	−160.8 (4)
C12A—Fe1—C25A—C21A	−48.7 (6)	C22B—Fe2—C25B—C23B	81.2 (4)
C11A—Fe1—C25A—C21A	−85.1 (4)	C14B—Fe2—C25B—C23B	−78.0 (4)
C15A—Fe1—C25A—C24A	112.2 (4)	C23B—Fe2—C25B—C21B	−118.1 (6)
C14A—Fe1—C25A—C24A	71.3 (4)	C15B—Fe2—C25B—C21B	122.9 (4)
C21A—Fe1—C25A—C24A	−119.6 (5)	C24B—Fe2—C25B—C21B	−80.1 (4)
C22A—Fe1—C25A—C24A	−81.4 (4)	C13B—Fe2—C25B—C21B	−166.3 (6)
C23A—Fe1—C25A—C24A	−38.0 (3)	C12B—Fe2—C25B—C21B	51.2 (8)
C13A—Fe1—C25A—C24A	34.7 (12)	C11B—Fe2—C25B—C21B	81.1 (4)
C12A—Fe1—C25A—C24A	−168.3 (4)	C22B—Fe2—C25B—C21B	−36.9 (4)
C11A—Fe1—C25A—C24A	155.3 (3)	C14B—Fe2—C25B—C21B	163.9 (3)
C15A—C11A—C31A—C32A	−145.6 (5)	C15B—C11B—C31B—C36B	−145.8 (5)
C12A—C11A—C31A—C32A	34.9 (7)	C12B—C11B—C31B—C36B	32.1 (8)
Fe1—C11A—C31A—C32A	124.6 (4)	Fe2—C11B—C31B—C36B	−56.3 (6)
C15A—C11A—C31A—C36A	36.6 (7)	C15B—C11B—C31B—C32B	33.6 (8)
C12A—C11A—C31A—C36A	−142.9 (5)	C12B—C11B—C31B—C32B	−148.5 (5)
Fe1—C11A—C31A—C36A	−53.2 (6)	Fe2—C11B—C31B—C32B	123.1 (5)
C36A—C31A—C32A—C33A	5.5 (7)	C36B—C31B—C32B—C33B	1.2 (7)
C11A—C31A—C32A—C33A	−172.3 (5)	C11B—C31B—C32B—C33B	−178.2 (5)
C36A—C31A—C32A—C1A	−171.5 (5)	C36B—C31B—C32B—C1B	−173.6 (5)
C11A—C31A—C32A—C1A	10.7 (7)	C11B—C31B—C32B—C1B	7.0 (7)
O1A—C1A—C32A—C31A	−123.4 (5)	O1B—C1B—C32B—C33B	−123.3 (5)
N1A—C1A—C32A—C31A	59.7 (6)	N1B—C1B—C32B—C33B	56.5 (6)
O1A—C1A—C32A—C33A	59.4 (6)	O1B—C1B—C32B—C31B	51.5 (7)
N1A—C1A—C32A—C33A	−117.5 (5)	N1B—C1B—C32B—C31B	−128.6 (5)
C31A—C32A—C33A—C34A	−5.3 (8)	C31B—C32B—C33B—C34B	−1.7 (8)
C1A—C32A—C33A—C34A	172.0 (5)	C1B—C32B—C33B—C34B	173.2 (5)
C32A—C33A—C34A—C35A	1.1 (8)	C32B—C33B—C34B—C35B	0.5 (8)
C33A—C34A—C35A—C36A	2.7 (8)	C33B—C34B—C35B—C36B	1.2 (8)
C34A—C35A—C36A—C31A	−2.4 (8)	C34B—C35B—C36B—C31B	−1.7 (8)
C32A—C31A—C36A—C35A	−1.7 (8)	C32B—C31B—C36B—C35B	0.5 (8)
C11A—C31A—C36A—C35A	176.1 (5)	C11B—C31B—C36B—C35B	179.9 (5)

### Hydrogen-bond geometry (Å, °)

**Table:**

Cg1 and Cg2 are the centroids of the C31A–C36A, C31B–C36B rings, respectively.

**Table:**

*D*—H···*A*	*D*—H	H···*A*	*D*···*A*	*D*—H···*A*
N1A—H1A···N6B	0.88	2.24	3.113 (5)	172
N1B—H1B···N6A	0.88	2.14	3.004 (5)	167
C3A—H3A···O1A	0.95	2.26	2.853 (7)	119
C3B—H3B···O1B	0.95	2.28	2.839 (7)	117
C7B—H7B2···O1B^i^	0.98	2.60	3.050 (6)	108
C7A—H7A1···Cg1	0.98	2.78	3.688 (7)	154
C7B—H7B1···Cg2	0.98	3.68	3.490 (7)	140

Symmetry codes: (i) −*y*+1/3, −*x*+2/3, *z*+1/6.
